# Supplementary material for: Content-rich biological network constructed by mining PubMed abstracts
Source: BMC Bioinformatics. 2004 Oct 8;5:147. doi: 10.1186/1471-2105-5-147 (PMC528731; doi:10.1186/1471-2105-5-147)
Supplement: Additional File 2 — The original results of the above study (non-essential files are deleted to keep the file size under the limit set by BMC bioinformatics). [file 1471-2105-5-147-S2.bz2 › chilibotAdditionalFile2/dip10/7ID8999959E17/html/TCF4_ID3.html]

 


 **TCF4** and **ID3** 
  
Found 7 abstracts in PubMed, retrieved 7.  
 

 What does Google say? 
 PDF only 
| .edu only 

---

**Interactive relationship** (e.g. stimulation, inhibition, etc)

**Inhibitory relationship**- We have used an interaction cloning strategy to identify an inhibitory isoform of the ITF 2  [ **TCF4** ]  transcription factor, ITF 2b, that interacts with the transcriptional inhibitor  **Id3**  HLH462.  Ref: 8999959 J Biol Chem, 1997
**Neutral relationship**- Additionally,  **Id3**  shows evidence of interacting with the related proteins E2 2  [ **TCF4** ]  and MyoD, but not c Myc.  Ref: 8759016 Nucleic Acids Res, 1996

**Non-interactive relationship** (e.g. studied together, co-existance, homology, etc.)

- Dimerization of three Id proteins Id1, Id2, and  **Id3**  with the four class A E proteins E12, E47, E2 2  [ **TCF4** ] , and HEB and two groups of class B proteins, the myogenic regulatory factors MRFs MyoD, myogenin, Myf 5 and MRF4 Myf 6, and the hematopoietic factors Scl Tal 1, Tal 2, and Lyl 1 were tested in a quantitative yeast 2 hybrid assay.  Ref: 9242638 J Biol Chem, 1997
